# Isolated giant renal hydatid cyst with a simple renal cyst appearance: a case report

**DOI:** 10.1186/s13256-022-03630-1

**Published:** 2022-10-24

**Authors:** Mohammed Hammade, Sami Alhoulaiby, Adnan Ahmed

**Affiliations:** 1 Faculty of Medicine, Damascus University, Damascus, Syria; 2 Al-Assad University Hospital, Damascus University, Damascus, Syria

**Keywords:** Isolated renal hydatid cyst, Renal echinococcosis

## Abstract

**Background:**

Isolated renal hydatid cysts of the kidney are a rare occurrence that account for about 2–3% of all hydatidoses. They can stay asymptomatic for years and could have a variable presentation on imaging techniques, which results in a challenging diagnostic process.

**Case presentation:**

We report a 22-year-old Caucasian male with a large cyst on the upper pole of the left kidney that had no septations nor membrane calcifications on computed tomography, which led to mistakenly considering it a simple renal cyst. The true diagnosis was identified intraoperatively and proven postoperatively by pathology.

**Conclusions:**

This case highlights the importance of keeping echinococcosis in mind when treating suspected renal cysts and tumors to avoid incorrect treatment and possible content spillage, anaphylaxis, and peritoneal dissemination.

## Background

Cystic echinococcosis (hydatidosis), a parasitic disease caused by the larval form of *Echinococcus granulosus*, is a common endemic disease in sheep-breeding countries worldwide, where sheep, dogs, and humans live in close contact [1]. In this cycle, humans are an accidental intermediate host through ingestion of *Echinococcus* eggs shed in feces. The liver is the most involved organ, followed by the lungs, which together account for 90% of cases, while renal echinococcosis is an unusual location in only 2–3% of infections [2]. Although a concurrent localization of renal cysts along with hepatic and/or pulmonary cysts is probable and facilitates the diagnosis, an isolated presentation is hard to differentiate from other renal lesions, as they could cover up to 32% of inspected renal masses [3]. Furthermore, renal hydatid cysts typically remain asymptomatic for years, and therefore grow enormously before being detected after presentation with a constellation of possible symptoms including flank pain, hematuria, pyuria, or intermittent fever [4].

An additional challenge posed by the ambiguity of this presentation is rupture of the iatrogenic cyst, and the consecutive acute abdomen with a 6% mortality rate [5]. This case has been reported in line with the CARE guidelines [6]. Renal hydatid cysts may present with various clinical findings, ranging from asymptomatic clinical course to total loss of renal function. It is beneficial to consider a renal hydatid cyst in patients when treating suspected renal cysts and tumors, especially in societies where hydatid cyst (HC) disease is endemic, even with negative immunological results.

## Case presentation

A 22-year-old Caucasian male farmer presented to our surgery clinic with hypogastric discomfort that started 1 month earlier and was not accompanied by severe pain, radiation of pain, nausea, vomiting, burning micturition, flank pain, pyuria, hematuria, or fever. Similarly, he had an insignificant surgical and medical history. His physical examination was normal except for mild tenderness in the left hypochondrium. There were no abnormal pulmonary signs and no peripheral lymphadenopathy.

Complete blood count, renal function tests, and liver function tests were normal with no eosinophilia. Immunological examination revealed negative Echinococcus immunoglobulin (Ig)G antibody titers (reference: negative ≤ 1:160). Urine tests did not reveal any signs of hydatiduria, and chest X-ray was also normal.

**Fig. 1:**
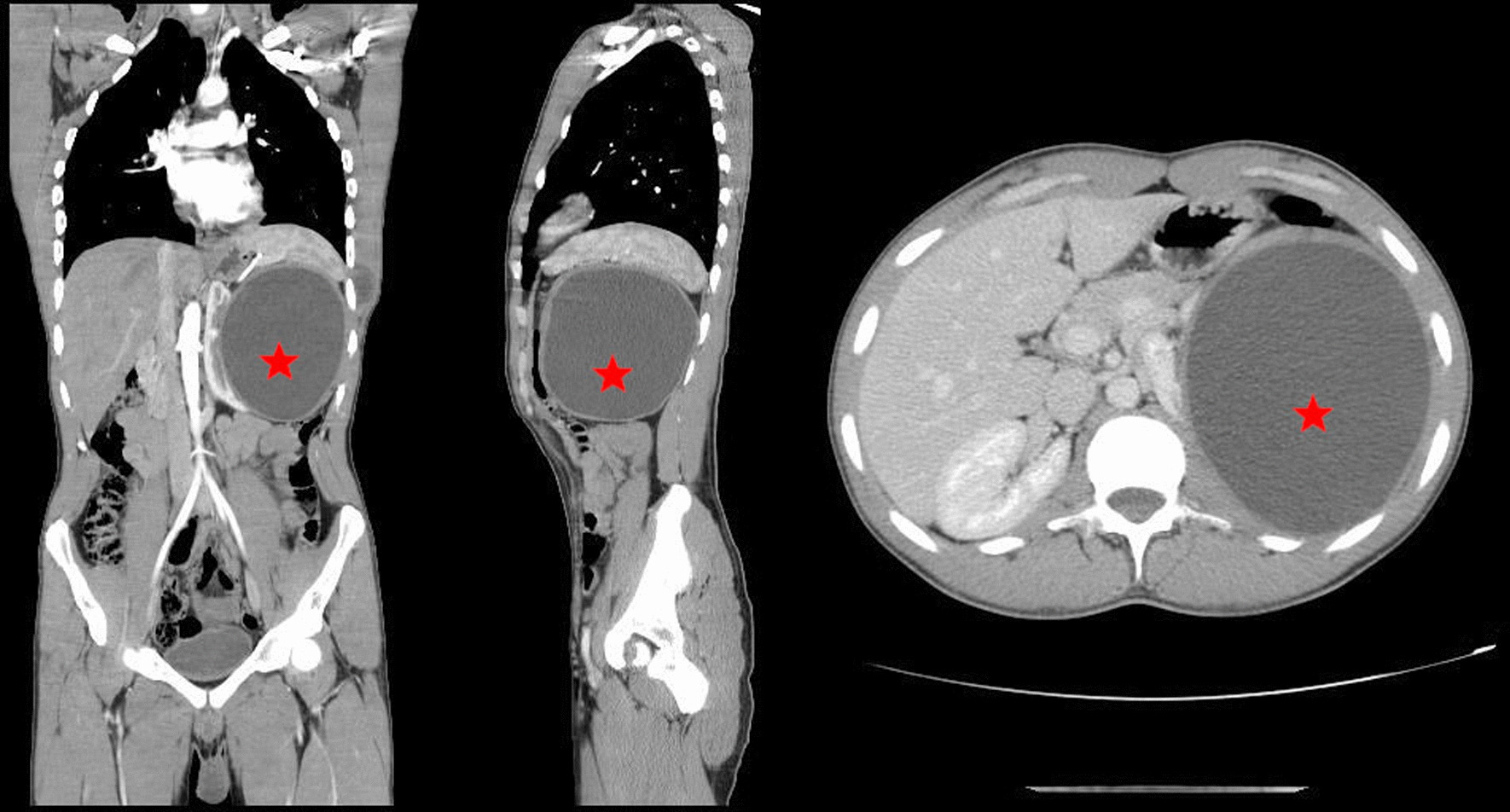
Contrast-enhanced CT (CECT) of the abdomen showing a large exophytic cyst (star) measuring 14 × 13.5 × 14 cm in the middle and upper pole of left kidney with no solid component or calcifications. Taken in all sections: coronal (left), sagittal (middle), and transverse (right)

Ultrasonography of the abdomen and pelvis revealed a well-defined lesion in the mid and upper pole of the left kidney, measuring 14 cm in the largest dimension. Contrast-enhanced computed tomography (CECT) of the abdomen and pelvis confirmed these findings, and showed a compression of the kidney downwards, anteriorly, and medially, as well as an upwards pushed spleen. The cyst appeared to have a thickened, nonenhancing, noncalcified regular wall surrounding a homogeneous fluid. No similar lesion in the contralateral kidney, liver, nor lungs could be detected (Fig. 1).

**Fig. 2:**
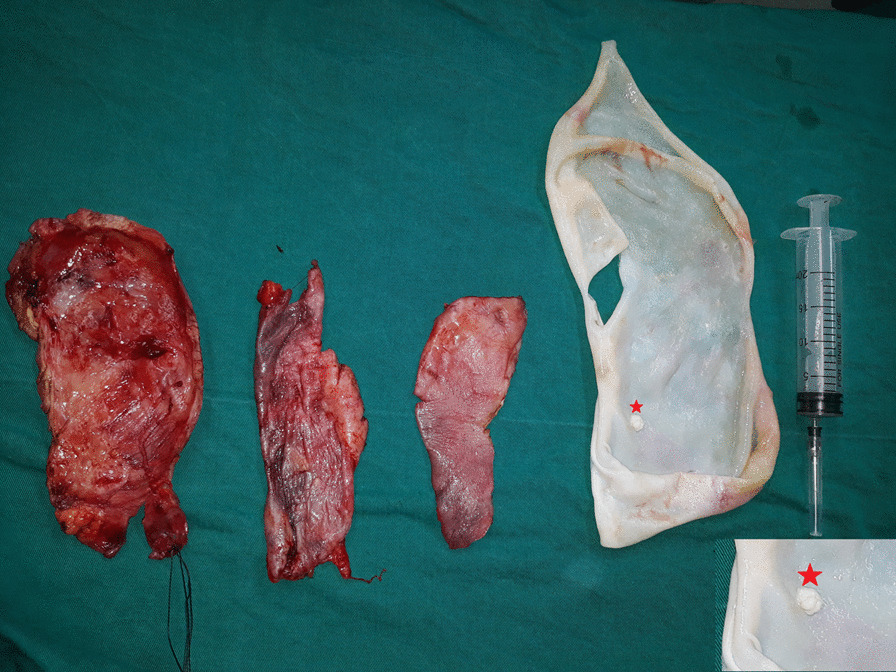
Postoperative surgical image; germinal membrane (right) with a daughter cyst is depicted in the insert (star, right) and parts of the cystic wall (left)

With these findings, a clinical diagnosis of a simple renal cyst was made, with a suspicion of renal hydatid disease. A transabdominal retroperitoneal approach through a subcostal incision was planned, with the aim of organ preservation. During the exploration, a large cystic structure was found to arise from the middle and the upper pole of the left kidney. After sucking the inner cyst fluid, we strongly suspected a hydatid disease; therefore, the operation field was protected with swabs soaked in 20% hypertonic saline. The cyst was opened and contents were evacuated, and 20% hypertonic saline was instilled as a scolicidal agent owing to the high probability of an uncontrolled spillage of content. Finally, the cyst was excised with a rim of normal parenchyma, and the specimen was sent for pathological examination (Fig. 2).

**Fig. 3:**
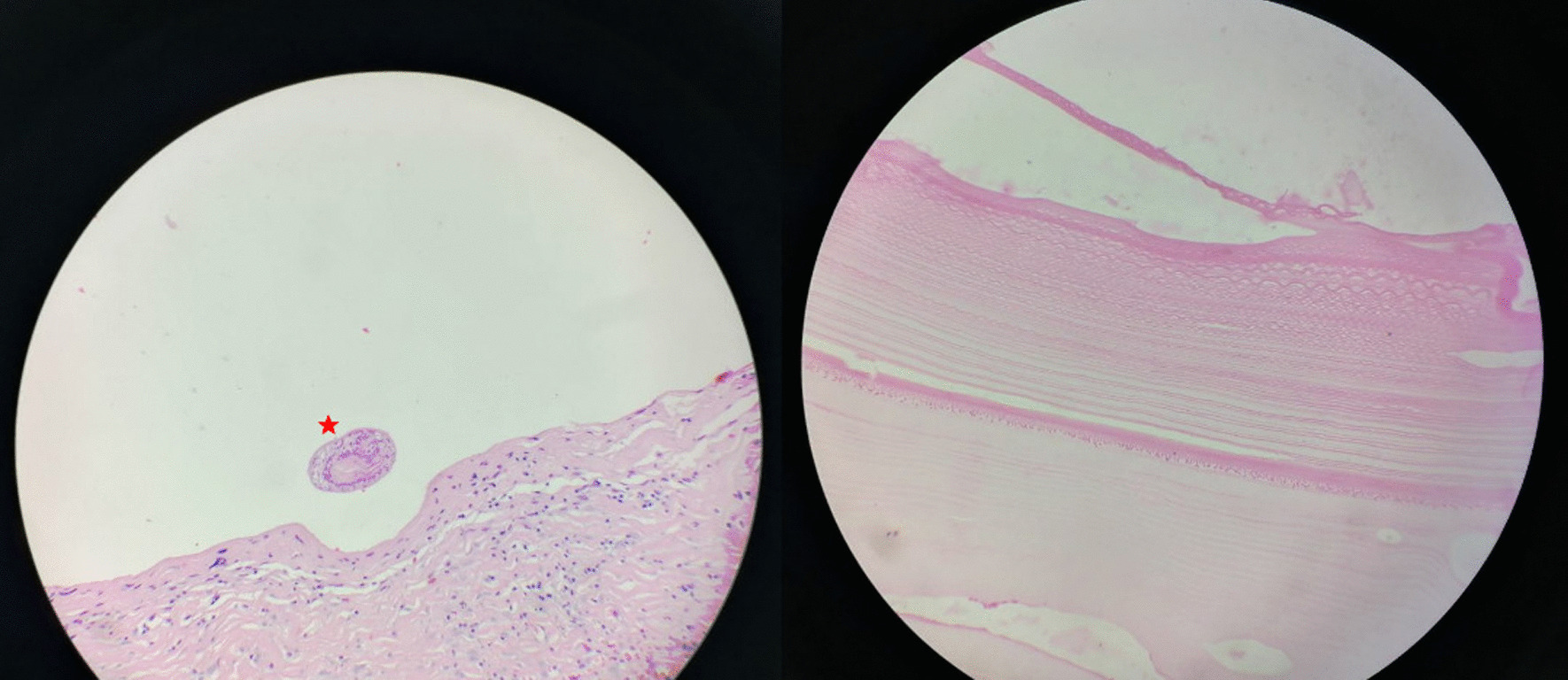
Histopathology image showing acellular laminated hydatid cyst membrane (right) and a scolex (star, left)

Histopathologic examination confirmed the hydatidosis with the presence of a laminated acellular and eosinophilic membrane along with many scolices in the cystic lumen (Fig. 3).

The patient was discharged on the third postoperative day with oral albendazole 400 mg for 3 months. No recurrence was detected in the postoperative 3- and 6-month follow-up ultrasound (USG) and 6-month follow-up computed tomography (CT) scan, and the patient remained in good health with normal renal function.

## Discussion

In this study, we report a 22-year-old male with a large cyst on the upper pole of the left kidney that had no septations nor membrane calcifications on CT-scan, which led to mistakenly considering it a simple renal cyst. The true diagnosis was guessed intraoperatively and proven postoperatively by pathology. Renal hydatid cysts may present with various clinical findings, ranging from asymptomatic clinical course to total loss in renal function. It is beneficial to keep echinococcosis in mind when treating suspected renal cysts and tumors.

Echinococcosis is a common endemic disease that presents with cysts in different organs, predominantly the liver and the lungs, with a rare settlement elsewhere. It could be a problematic disease to cure when it disseminates [2]. In the seldom cases of renal localization, the cyst is often located in the upper pole (37% of reported cases) [4]. The ages of the affected patients ranged between 30 and 50  years [21], while, perhaps surprisingly, renal hydatid disease is extremely rare in the pediatric population, accounting for approximately 1.9% of all cases [19, 20]. However, a high suspicion of this disease should be considered in any cystic mass of the kidney in children, especially in endemic regions. Diagnostic and therapeutic methods conform with those applied to hepatic and pulmonary hydatidosis, including ultrasonography, serology, and CT, in addition to postoperative histopathology [3].

However, the isolated occurrence of renal hydatid cysts presents a diagnostic challenge, as the clinical and imaging findings could either be understated and the cyst considered a simple renal cyst, especially with the absence of internal septation and mural calcifications, or on the contrary, overstated and feared to be malignant when it has a lobulated appearance [7, 8]. Others also misinterpreted the findings for an abscess [9]. In this case, the patient presented with a cyst void of septations and membrane calcifications on CECT, and the absence of positive Echinococcus immunoglobulin (Ig)G antibody titers, as well as hydatiduria, led to a misdiagnosis of a simple renal cyst. Only after the pathology report were we able to establish a specific diagnosis.

Treatment is modulated based on different factors. Conservative treatment requires a confident diagnosis, which was unavailable. Percutaneous management (puncture, aspiration, injection of scolicidal agent, reaspiration) might be a safe option [10]. However,there are some disadvantages to this method, including dissemination of daughter cysts and fatal anaphylaxis [11, 12]. Surgical treatment is the other option and could take the form of complete excision of the cyst with pericystectomy, or partial/complete nephrectomy depending on the residual functional parenchyma. This requires extreme caution to avoid spillage, recurrence, or the development of severe anaphylactic shock [13].

Although laparoscopy, transperitoneal and retroperitoneal, has been reported in cases of hydatid renal cysts [14, 15], such procedures can only be performed at centers with vast experience in laparoscopic surgery. An open surgical approach is preferred at developing centers where laparoscopic expertise is not available, as in our case. Based on our findings, we went for a transabdominal retroperitoneal approach. Postoperatively, we gave the patient prophylactic albendazole as per international recommendations [16].

It is worth noting that during the last decade, the number of urological laparo-endoscopic single-site (LESS) procedures performed worldwide has increased. Symeonidis *et al*. reported that in the hands of experienced surgeons, LESS seems a feasible, efficient, and less invasive alternative to standard/conventional laparoscopy [17]. Moreover, Zouari *et al*. assessed the feasibility and outcomes of pediatric LESS, successfully treating one renal hydatid cyst (RHC) case [18]. There is no doubt that if this endoscopic technique is applied in the management of RHC in adults, it may have some advantages, although this depends on many factors and perhaps future studies can evaluate this.

## Conclusions

Isolated hydatidosis of the kidney is a rare entity that could easily be confused with renal cysts, malignancies, and abscesses, thus it is important to keep in mind as a crucial differential diagnosis in such cases to avoid incorrect surgical intervention, and possible content spillage, anaphylaxis, and peritoneal dissemination.
